# Physical and Psychosocial Functions of Adults with Lower Limb Congenital Deficiencies and Amputations in Childhood

**DOI:** 10.1155/2016/8109365

**Published:** 2016-04-18

**Authors:** Ll. Montesinos-Magraner, D. Issa-Benítez, E. Pagès-Bolíbar, M. Meléndez-Plumed, M. A. González-Viejo, C. Castellano-Tejedor

**Affiliations:** ^1^Department of Rehabilitation and Traumatology, University Hospital Vall d'Hebron, Passeig de la Vall d'Hebron 119-129, 08035 Barcelona, Spain; ^2^Department of Psychiatry, University Hospital Vall d'Hebron, Passeig de la Vall d'Hebron 119-129, 08035 Barcelona, Spain; ^3^Vall d'Hebron Research Institute, Passeig de la Vall d'Hebron 119-129, 08035 Barcelona, Spain

## Abstract

*Objectives.* (1) To describe the epidemiological and medical features of a sample with LLA and LLD in childhood and (2) to explore their relationship with subsequent physical and psychosocial functions in adulthood.* Methods.* Cross-sectional survey. Demographics, medical data, Locomotor Capabilities Index (LCI), and Discomfort-Engagement in Everyday Activities Involving Revealing the Body Scale (D-EEARB) were collected from thirty-two adults who suffered from LLA in childhood or LLD.* Results.* Most of the sample (53.1% males) was working (84.4%), living independently (75%), and single (75%). Mean age was 33.16 (SD = 7.64, range 18–50). Leading causes for LLA were traumatic (40.6%) and oncologic (25%). LLD was present in 6 cases (18.8%). LCI scores revealed a high performance among males (*t*_17,464_ = 2.976, *p* = .008). D-EEARB scores showed that 56.25% stated feeling “quite” or “totally comfortable” in situations which involved revealing their body, but 43.75% stated the contrary (“uncomfortable” or “very uncomfortable”). LLD and traumatic LLA show higher scores in D-EEARB than vascular and oncological LLA (*χ* ^2^ = 7.744, df = 3, *p* = .05).* Conclusions.* Adults suffering from LLDs and LLAs during childhood seem to perform well once they are adults. However, 43.75% of patients express considerable discomfort in situations that involve revealing the body.

## 1. Introduction

Lower limb amputations (LLAs) and lower limb deficiencies (LLDs) in children can be due to congenital or acquired etiology. Congenital limb deficiency means that there is partial or total absence of the limb [1]. Regarding acquired limb amputations, the majority of them are due to trauma, mainly caused by power tools and machines, vehicular accidents, gunshot wounds, and explosions or electrical injuries [2]. More than 90% of traumatic amputations during childhood are unilateral and 60% occurred in the lower limb [2]. The third most frequent etiology is due to oncological tumours, followed by other diseases such as infection, vascular diseases, and neurological dysfunctions [3–5]. Demographic studies have established the preponderance of the congenital limb deficiencies (approximately 60%) compared to acquired limb amputations [2, 6]. The ratio of male children with limb deficiency is 3 : 2 compared to female [2, 6, 7]. LLA and LLD patients during childhood will require a large number of prosthetic replacements. Approximately, during the first 5 years of their life, they will require a replacement per year and a replacement every two years from the age of 5 to 12 years [7].

Psychological problems that can arise following amputation can be of diverse nature and severity. The majority of studies on adjustment to amputation are focused on traumatic amputations or oncologic but there are no studies comparing both samples and even LLD [8]. Moreover, they have not included in their analysis many key moments such as the immediate reaction to amputation, the rehabilitation period, and other moments related to the development of a changed body image, self, and identity. If amputees are children or adolescents, identity and socialization issues gain special interest [9–11].

Regarding the etiology, it is expected that unforeseen amputations (such as amputations due to severe traumas) could be related to higher anxiety, depression, frustration, or hostility [8, 12]. On the one hand, congenital limb deficiency patients grow with this absence and they do not usually perceive the amputation as a disability, a disease, or a traumatic event [8, 13]. Therefore, the rehabilitative treatment should have as an objective the improvement of the functionality, but not necessarily the replacement of the deficiency with lower limb prosthetic fitting. Scientific literature in this field has described that, due to the absence of an acute trauma leading to the amputation, a better acceptance and adjustment is expected [8]. A child born with a limb deficiency does not feel a sense of loss (either loss of sensation or function) because this is the only body he/she has ever known [13]. However, other studies have stressed that body functions could be limited due to the limb deficiency and children could feel hampered to successfully perform a different range of normative age-developmental activities such as rolling, crawling, grasping, and exploring. On the other hand, noncongenital amputees feel the amputation as a traumatic event that might lead to a process of loss and grief [14, 15]. Some studies have described emotions such as grief and bereavement among traumatic LLA, similar to experiencing the death of a loved one [16, 17]. In this sense, patients suffering from a traumatic LLA will have to cope with the loss of sensation from the missing limb, the loss of function, and the changes in the body image as well as other people's perception of his/her body image [18]. Additionally, some patients could show difficulties in accepting their deficiency and develop certain dependency on others (e.g., parents and health professionals). Such dependencies could have detrimental effects on rehabilitative work and, consequently, on the entire physical and psychological adaptation process [19–21].

Lower limb prosthetic fitting in childhood serves functional purposes and is closely related to psychomotor development, in which children first start to stand, then to walk, and later to do all other different activities (e.g., to run and to play). Thus, it should start as soon as the child initiates standing up [2, 7, 22]. Once they are adolescents, the prosthesis will not only serve functional purposes but also play an important role in socialization and self-identity processes (e.g., to practice sports or other social activities with peers). Younger amputees without previous history of pathologies or emotional problems show better adjustment to the prosthesis than adolescents or young adult amputees [8]. Since the prosthesis serves to play, to have social interaction, and to overcome physical limitations imposed by the limb deficiency, they internalize it more easily and have a better disposition to accept the fitting [8].

Children with LLA or LLD, when grown adults, will have to face different physical and psychosocial challenges that might influence their participation and quality of life [21]. Unfortunately, there are few studies examining psychosocial consequences of LLA and LLD amongst this population. One review study [8] highlighted this understudied area of research and showed evidences that psychosocial functioning and social discomfort might be increased in the postacute period of the amputation and 10–20 years after amputation.

For these reasons, the present study has two main objectives: (1) to describe the epidemiological and medical features of LLA and LLD in childhood and (2) to explore the relationship of such features with the physical and psychosocial functions of this population once they are adults. Given the exploratory nature of the study, there are no formal hypotheses. However, it is expected to observe higher physical and psychosocial outcomes among congenital LLD compared to LLA regardless of gender.

## 2. Methods

### 2.1. Study Design

Cross-sectional surveys were conducted.

### 2.2. Participants

Participants were recruited from a patients' database at the Orthotics and Prosthetics Department of the rehabilitation outpatient clinic at the University Hospital Vall d'Hebron (Barcelona, Spain), a referral hospital of tertiary level. Recruitment and data collection were conducted between October 2007 and October 2008. Study's inclusion criteria were as follows: (1) patients are treated and with follow-up visits at the referenced hospital throughout the prosthetic process, (2) there is unilateral lower limb amputation or congenital lower limb deficiency, (3) in case of LLA, this must have occurred before 18 years of age, and (4) patients' age at assessment is more than 18 years. Exclusion criteria were as follows: (1) bilateral lower limb amputation or deficiency, (2) upper limb amputation, (3) younger than 18 years old at assessment, and (4) individuals diagnosed with severe cognitive impairment (e.g., traumatic brain injuries) unable to comprehend study materials.

### 2.3. Procedure

Patients were recruited from the hospital's database of patients treated in the Orthotics & Prosthetics Clinic during the aforementioned period of time. All participants were approached by phone by only one rehabilitation medical doctor (main researcher of this study). In this first contact, the professional explained the objectives of the study and asked for participation. If the patient agreed to participate, informed voluntary consent was requested. Next, demographics and medical data were collected. Measures of functional outcomes (Locomotor Capability Index parts I and II; LCI-I and LCI-II) [23, 24] and psychosocial functionality (Discomfort Related Engagement in Everyday Activities Involving Revealing the Body Scale; D-EEARB) [25] were also obtained via semistructured interview. All data was collected in a 45-minute telephone interview by the same professional (a trained rehabilitation physician and main researcher of this study).

### 2.4. Ethical Aspects

This research complies with the Ethical guidelines of Hospital Vall d'Hebron and Vall d'Hebron Research Institute with the Helsinki Code. All patients participated on a voluntary basis. Anonymity was guaranteed in the first contact, and the main researcher of this study disclaimed that data would be exclusively used for the purposes of the present research.

### 2.5. Assessment Tools

#### 2.5.1. Sociodemographic and Medical Data

Sociodemographic (age, gender, employment status, emancipation, and relationship status) and medical data (etiology of the amputation, level of the amputation, laterality, secondary complications, age of amputation, and number of prosthesis replacements) were obtained via a self-reported ad hoc questionnaire and later completed with medical records.

#### 2.5.2. Locomotor Capability Index Parts I and II (LCI-I and LCI-II)

To assess the goals and achievements of patients receiving prostheses, the LCI [23, 24] was administered. The LCI is a self-administered scale and is widely used to assess a patient's perceived capability to perform 14 different locomotor activities while wearing prosthesis, selected from the Locomotor Disabilities Classification of the World Health Organization [26]. Each item is scored in a 4-point ordinal scale (ranging from 0 =* not able* to 3 =* able to accomplish the activity alone*). A summary score of the global locomotor ability level is obtained by adding the individual scores assigned to each activity for a possible maximum of 42 points. Additionally, the LCI can be divided into two 7-item subscales that cover “basic activities/skills” such as “Walk in the house” and “Step down a sidewalk curb” (items 1, 4, 5, 8, 9, 10, and 11) and “advanced activities/skills” such as “Walk outside in inclement weather (e.g., snow, rain, and ice)” and “Walk while carrying an object” (items 2, 3, 6, 7, 12, 13, and 14). Higher scores reflect better locomotor capabilities with the prosthesis and less dependence on assistance. The LCI was found to have good reliability and validity [23, 24, 27]. In this study, Cronbach's Alpha was 0.95.

#### 2.5.3. Discomfort Related Engagement in Everyday Activities Involving Revealing the Body Scale (D-EEARB)

The D-EEARB is an 11-item scale requiring respondents to imagine they are undertaking each of the activities in the scale which involve revealing their body to others (general others, 8 items; partner, 3 items) and rate how comfortable they would feel in these situations (plus one additional situation which involves going to the beach). The response options range from 1 =* very uncomfortable* to 4 =* totally comfortable*, and total scores range between 11 and 44 points, with higher scores indicating higher comfort while revealing the body.

The D-EEARB was found to have good reliability and validity [25]. In the present study, Cronbach's Alpha was 0.91.

### 2.6. Statistical Analyses

Statistical analyses were performed using SPSS 19.0. Statistical significance was assumed for *p* values < .05. Descriptive statistics were performed to describe the sample and to calculate LCI (LCI total, LCI-I, and LCI-II) and D-EEARB scores. Correlation analyses, two-sample *t*-tests for continuous variables, and chi-square tests for categorical variables were used to compare differences between groups (considering all the medical and demographical variables included in this study). Size effects were included (IC 95% and Cohen's *d*) when significant differences were found. There were no missing values in the present research.

## 3. Results

From an initial pool of 45 potential candidates to participate in the study, 33 were successfully approached and 12 were not reached (e.g., not answering the phone or wrong contact information). The final sample consisted of 32 patients since one of the initial 33 finally declined to participate arguing lack of time to answer the questions. All of them gave informed consent prior to answering the survey. Demographics and medical data of the sample are shown in Table 1.

Most of the assessed samples were working and single, and more than a half of them lived independently. The most frequent etiology for amputation was traumatic accidents (including traffic accidents and, in two cases, electrical accidents and burns. The second leading cause of the amputation was oncologic), specifically osteosarcomas (see Table 1 for details). Only 6 cases suffered from a congenital LLD.

The level of amputation was distributed into three main groups: hip disarticulation/transfemoral amputation, knee disarticulation/transtibial amputation, and foot level. Finally, secondary complications were present in 50% of the sample. Bone overgrowth occurred in 6 patients and among them, only one experienced phantom pain. No patients developed local infection and all of them used prosthesis (see Table 1 for details).

### 3.1. Functional Results

Locomotor capabilities' scores (LCI) were high (*M* = 40.22, SD = 2.73, median = 41.50, and range = 33–42), scoring higher than 33 points. Specifically, 51.2% (*n* = 23) scored 41 or 42, the maximum value of the LCI.

The LCI-I scores were also high (*M* = 20.12, SD = 1.60, median 21, and range = 16–21); 68.8% of the sample (*n* = 22) achieved the maximum score of 21 and 5 patients (15.6%) scored ≤ 18 points.

In the LCI-II (*M* = 20.09, SD = 1.97, median 21, and range = 12–21), 84.4% of the sample (*n* = 27) scored 20 or 21 (maximum scoring).

No statistically significant relationship was observed between age at the assessment, age at amputation, and years since the amputation with any of the LCI scores. However, gender was significantly related to the LCI total scores (*t*_17.464_ = 2.976, *p* = .008, *d* = 1.08, and IC 95% 0.781–4.560) and the LCI-II scores (*t*_14.540_ = 2.247, *p* = .041, *d* = 0.81, and IC 95% 0.076–3.038), with males showing higher scores (see Table 2).

Number of prosthesis replacements and secondary complications showed no significant relationship with any of the LCI scores. Finally, the etiology of the amputation was significantly related to the LCI-II scores (*χ* ^2^ = 11.22, df = 3, *p* = .011). In this sense, congenital (*M* = 21.00, SD = 0.00, median = 21.00, and range 19–21) and traumatic etiologies (*M* = 20.77, SD = 0.6, median = 21.00, and range 19–21) showed higher scores than vascular (*M* = 19.80, SD = 2.17, median = 21.00, and range 16–21) and oncologic etiologies (*M* = 18.5, SD = 3.07, median = 20.00, and range 12–21).

### 3.2. Psychosocial Results

According to the results shown in the D-EEARB scale, 56.25% of the sample (*n* = 18) stated that they feel “quite comfortable” or “totally comfortable” in the situations described in the scale (scores ≥ 33 points). 43.75% of the sample (*n* = 14) expressed being “uncomfortable” or “very uncomfortable” in the majority of situations described in the scale, scoring ≤29 points.

With regard to the medical variables considered in the study, only one significant relationship was found regarding the etiology of the amputation and the D-EEARB scores (*χ* ^2^ = 7.744, df = 3, *p* = .05). In this sense, congenital (*M* = 40.83, SD = 3.37, median = 41.50, and range 36–44) and traumatic etiologies (*M* = 34.38, SD = 9.32, median = 33.00, and range 20–44) showed higher mean scores than vascular (*M* = 26.80, SD = 9.88, median = 21.00, and range 18–39) and oncologic etiologies (*M* = 30.00, SD = 7.67, median = 28.00, and range 20–44).

The only demographic variable that was significantly related to the D-EEARB scores was the relationship status (*t*_25.89_ = 4.199, *p* < .001,  *d* = 3.04, and IC 95% 5.061–14.772), with those being single showing higher mean scores, that is, feeling more comfortable in everyday activities that involve revealing the body (*M* = 40.75, SD = 4.23 versus *M* = 30.83, SD = 8.95), see Table 3.

## 4. Discussion

The most frequent causes for LLA in the studied sample were traumatic (40.6%) and oncologic (25%) [1, 3, 6]. In contrast with the revised epidemiological studies, a lower rate of congenital LLD was found. As referred to by Al-Worikat and Dameh [6], this could be explained because congenital LLD usually happens in distal extremity areas, such as fingers, that might involve less disability and, consequently, these patients are not necessarily treated in highly specialized hospitals. In our case, gender was evenly balanced (53.1% men) as well as lower limb laterality (right 56.3%), and we did not find the higher prevalence of males suffering from LLD or LLA as suggested in other studies [2–6].

Scarce scientific literature exists on secondary complications after LLA or LLD during childhood [3, 6, 8]. However, Melzack et al. [28] have described that oncologic amputees in early childhood have higher risk of suffering from phantom limb pain than traumatic LLA or congenital LLD. In our sample, secondary complications seemed to be infrequent and mainly related to local pain (15.6%) or bone overgrowth (18.7%). The only patient who suffered from phantom limb pain was, precisely, a LLA patient due to oncologic cause.

Contrary to what has been indicated in some studies, a high percentage of the sample studied (84.4%) was working and living independently (65.6%). Some authors [29, 30] have stressed that sometimes LLA and LLD patients could be seen as “disabled” and part of a “stigmatized” group in the eyes of people who do not have limb deficiencies. This assumption could lead to people suffering disabilities to experience feelings of inability or unfitness to perform regular adult activities such as work, live on their own, or create a family. Besides, one study has found that more than one-half of their studied sample of young LLAs visited friends and relatives less frequently since their amputation [31] and, additionally, two-thirds were less likely to go to the cinema, theatre, sport events, or other cultural and leisure activities [32, 33]. These last results could explain the high rate of single patients (suffering from both LLD and LLA) that we have found in our sample (75%). By restricting social participation, opportunities to start and establish a romantic relationship are reduced.

With regard to the LCI, high and satisfactory mean scores have been observed in the studied sample. High scores in the LCI show lower disability, better mobility walking with prosthesis during gait, and less need of assistance from a caregiver. We have not found significant relationships between the LCI scores and most of the medical variables considered (e.g., secondary complications, number of prosthesis replacements, and level of the amputation). The etiology of the amputation was revealed to be significant. In this sense, congenital LLD and traumatic LLA showed higher scores compared to the vascular and oncologic LLAs. We believe these results are relevant since there are not too many studies exploring the impact that different etiologies could entail on functional outcomes. However, the scarce existing literature exploring such issues points out to better general adjustment in congenital samples due to reduced comorbidities [7, 8, 13, 34]. Norvell et al. [35] showed LCI mean scores of 33 in adults with vascular pathology with transfemoral amputation and LCI mean scores of 40 in transtibial amputations. However, these relationships cannot be observed in the present study. This could be explained because the mean age at amputation in the studied sample was very low (8.67 years) and most of the patients have lived with prosthesis from the very first part of their childhood.

The most common psychological adjustment problems among adults who have suffered LLD or undergone LLA include depression, anxiety, low self-esteem, loss of a sense of wholeness, and social isolation. However, social discomfort and body-image anxiety have also been found among some people with amputations, and these have been associated with increased activity restriction, depression, and anxiety [8, 36]. A longitudinal research has also found that significant levels of psychological morbidity and social isolation, which were present immediately after the amputation, were still apparent in over 40% of the sample at one- and two-year follow-up periods [25, 37]. In our study, 43.75% of the patients feel “uncomfortable” or “very uncomfortable” but it has not shown significant relationship to gender or level of amputation. However, a strong relationship with traumatic and congenital etiologies has been found, with this sample population stating better psychosocial adjustment than vascular or oncologic amputees. Therefore, as hypothesized, congenital LLD showed better overall adjustment, both physically and psychosocially. Additionally, traumatic LLAs have also showed satisfactory outcomes in physical and psychosocial adjustment, revealing very similar results to congenital LLDs. The fact that amputation occurred in early childhood (before 9 years of age) could make the prosthesis fitting process very similar to that experienced by congenital LLD. Thus, traumatic LLAs would not have lived the sense of loss of function and self-image as that described in young or adult LLAs from traumatic causes. Another possible reason that could explain these results is that oncological and vascular etiologies are linked to more complicated and longer processes compared to the congenital or traumatic etiologies. Consequently, the adaptation of the patient to the prosthesis and rehabilitation period could be worse tolerated.

Finally, the fact that single patients showed better scores on the D-EEARB could be explained because of the nature of the relationships they establish with others. In this sense, patients with a partner may feel uncomfortable when revealing their body due to the fear of being judged or not attractive to the partner. One of the main concerns expressed among this population is to be able to maintain the partner [36]. If they think they cannot maintain them, they could dislike or even be unpleasant to their sentimental partner; it is very likely that they will express greater feelings of discomfort than single amputees. Single patients that do not have that kind of bond or whose romantic relationships are more sporadic might disregard the importance of revealing their body and grant minor attention to that action [25]. Additionally, it cannot be ruled out that single patients could have remained single to avoid revealing their body and exposing themselves to a partner and hence possible negative feelings.

### 4.1. Study Limitations and Recommendations for Further Studies

In order to add to the knowledge and update the literature on functional and psychosocial adjustment to LLDs and LLAs during childhood, more longitudinal research with higher samples is needed. By doing this, it would be possible to further examine potentially related variables such as medical and other psychological variables (e.g., personality and coping). Moreover, we recommend that both quantitative and qualitative research designs should be used for a better research approach to this issue.

Finally, interviews carried out in this study were made telephonically and it cannot be ruled out that this could have hindered answering very intimate questions related to the D-EEARB. Moreover, telephone interviews offer less control to the researchers (e.g., cannot see people's reactions, facial expressions, and gestures) and can limit complexity of questions since some people could hang up because of long questions or show decreased attention or interest in the survey. However, telephone interviews also have advantages such as being cost and time effective and having wide geographic access. These latter aspects were specially appreciated in the context of our research considering that most patients lived far from the hospital and they have no follow-up scheduled at the time of the study.

## 5. Conclusions

This study adds to the knowledge and pointed out that psychosocial aspects are still hampered in adulthood. Considering our results, the etiology seems to play a major role in the long-term physical and psychosocial adaptation of LLDs and LLAs in childhood. Therefore, efforts to design and implement appropriate physical and psychosocial screenings during and after amputation seem necessary. Only by doing this would it be possible to better understand the situation and needs of these understudied populations and to design tailored interventions aimed at preventing and treating correctly potential mid- and long-term comorbidities.

## Figures and Tables

**Table 1:** Demographics and medical characteristics of the sample (*N* = 32).

	*n*	%
Gender		
Male	17	53.1
Female	15	46.9
Employment status		
Working	27	84.4
Not working	5	15.6
Living independently		
Yes	21	65.6
No	11	34.4
Relationship status		
In a relationship	8	25
Single	24	75
Etiology		
Traumatic	13	40.6
Oncologic	8	25
Congenital	6	18.8
Vascular/septic	5	15.6
Level of amputation		
Hip disarticulation/transfemoral amputation	14	44
Knee disarticulation/transtibial amputation	14	44
Foot level	4	12
Lower limb laterality		
Right	18	56.3
Left	14	43.8
Secondary complications		
No	16	50
Local pain	5	15.6
Fracture	1	3.1
Infection	0	0
Bone overgrowth (BO)	4	12.5
Phantom pain + BO	1	3.1
Infection + BO + fracture	1	3.1
Others	4	12.5

	Mean (SD)	Range

Age at assessment (in years)	33.16 (7.64)	18–50
Age at amputation (in years)	8.67 (5.89)	0–17.5
Time since amputation (in years)	24.48 (7.76)	11.92–50
Prosthesis replacements	9.34 (6.00)	0–30

**Table 2:** Functional results by gender.

Questionnaire	Gender	Mean (SD)	Range (min–max)	Median	*p*
LCI total	Male (*n* = 17)	41.47 (1.23)	37–42	42	.007
	Female (*n* = 15)	38.80 (3.28)	33–42	39	

LCI-I	Male (*n* = 17)	20.65 (1.22)	16–21	21	n.s.
	Female (*n* = 15)	19.53 (1.81)	16–21	20	

LCI-II	Male (*n* = 17)	20.82 (0.39)	20-21	21	.041
	Female (*n* = 15)	19.27 (2.66)	12–21	21	

n.s.: no statistically significant differences.

**Table 3:** Psychosocial results by gender.

Questionnaire	Gender	Mean (SD)	Range (min–max)	Median	*p*
D-EEARB	Male (*n* = 17)	35.88 (8.35)	20–44	38.00	n.s.
	Female (*n* = 15)	30.40 (9.26)	18–44	28.00	

n.s.: no statistical significant differences.

## Abbreviations

LLA: Lower limb amputee
LLD: Lower limb deficiency
LCI-I: Locomotor Capabilities Index part I
LCI-II: Locomotor Capabilities Index part II
D-EEARB: Discomfort Engagement in Everyday Activities Involving Revealing the Body Scale.

## References

[1] Day H. J. B. (1991). The ISO/ISPO classification of congenital limb deficiency. *Prosthetics and Orthotics International*.

[2] Yigiter K., Ülger Ö., Şener G., Akgogan S., Erbahçeci F., Bayar K. (2005). Demography and function of children with limb loss. *Prosthetics and Orthotics International*.

[3] Rijnders L. J. M., Boonstra A. M., Groothoff J. W., Cornel M. C., Eisma W. H. (2000). Lower limb deficient children in the Netherlands: epidemiological aspects. *Prosthetics and Orthotics International*.

[4] Bryant P. R., Pandian G. (2001). Acquired limb deficiencies in children and young adults. *Archives of Physical Medice and Rehabilitation*.

[5] Yigiter K., Ülger Ö., Şener G., Akdoğan S., Erbahçeci F., Bayar K. (2005). Demography and function of children with limb loss. *Prosthetics and Orthotics International*.

[6] Al-Worikat A. F., Dameh W. (2008). Children with limb deficiencies: demographic characteristics. *Prosthetics and Orthotics International*.

[7] American Academy of Orthopaedic Surgeons (1992). *Atlas of Limb Prosthetics: Surgical, Prosthetic, and Rehabilitation Principles*.

[8] Horgan O., MacLachlan M. (2004). Psychosocial adjustment to lower-limb amputation: a review. *Disability and Rehabilitation*.

[9] Clerici C. A., Ferrari A., Luksch R. (2004). Clinical experience with psychological aspects in pediatric patients amputated for malignancies. *Tumori*.

[10] Ferrari A., Clerici C. A., Spreafico F. (2002). Psychological support in children and adolescents with cancer when amputation is required. *Medical and Pediatric Oncology*.

[11] Teall T., Barrera M., Barr R., Silva M., Greenberg M. (2013). Psychological resilience in adolescent and young adult survivors of lower extremity bone tumors. *Pediatric Blood & Cancer*.

[12] Shukla G. D., Sahu S. C., Tripathi R. P., Gupta D. K. (1982). A psychiatric study of amputees. *The British Journal of Psychiatry*.

[13] Datta D., Selvarajah K., Davey N. (2004). Functional outcome of patients with proximal upper limb deficiency—acquired and congenital. *Clinical Rehabilitation*.

[14] Belon H. P., Vigoda D. F. (2014). Emotional adaptation to limb loss. *Physical Medicine and Rehabilitation Clinics of North America*.

[15] Furst L., Humphrey M. (1983). Coping with the loss of a leg. *Prosthetics and Orthotics International*.

[16] Parkes C. M. (1975). Psycho-social transitions: comparison between reactions to loss of a limb and loss of a spouse. *The British Journal of Psychiatry*.

[17] Rybarczyk B., Edwards R., Behel J. (2004). Diversity in adjustment to a leg amputation: case illustrations of common themes. *Disability and Rehabilitation*.

[18] Schulz M. (2009). Coping psychologically with amputation. *Vasa*.

[19] Greive A. C., Lankhorst G. J. (1996). Functional outcome of lower-limb amputees: a prospective descriptive study in a general hospital. *Prosthetics and Orthotics International*.

[20] Schoppen T., Boonstra A., Groothoff J. W., de Vries J., Göeken L. N., Eisma W. H. (2003). Physical, mental, and social predictors of functional outcome in unilateral lower-limb amputees. *Archives of Physical Medicine and Rehabilitation*.

[21] Williamson G. M., Schultz R., Bridges M. W., Behan A. M. (1994). Social and psychological factors in adjustment to limb amputation. *Journal of Social Behaviour and Personality*.

[22] González-Viejo M. A., Cohí-Riambau O., Salinas-Castro F. (2005). *Amputación de Extremidad Inferior y Discapacidad: Prótesis y Rehabilitación*.

[23] Franchignoni F., Orlandini D., Ferriero G., Moscato T. A. (2004). Reliability, validity, and responsiveness of the locomotor capabilities index in adults with lower-limb amputation undergoing prosthetic training. *Archives of Physical Medicine and Rehabilitation*.

[24] Grisé M.-C. L., Gauthier-Gagnon C., Martineau G. G. (1993). Prosthetic profile of people with lower extremity amputation: conception and design of a follow-up questionnaire. *Archives of Physical Medicine and Rehabilitation*.

[25] Donovan-Hall M. K., Yardley L., Watts R. J. (2002). Engagement in activities revealing the body and psychosocial adjustment in adults with a trans-tibial prosthesis. *Prosthetics and Orthotics International*.

[26] World Health Organization (2001). *International Classification of Functioning, Disability and Health*.

[27] Franchignoni F., Tesio L., Orlandini D., Miller W. C., Deathe A. B., Speechley M. (2002). Mobility scales for lower-prosthetic patient: the locomotor capabilities index. *Archives of Physical Medicine and Rehabilitation*.

[28] Melzack R., Israel R., Lacroix R., Schultz G. (1997). Phantom limbs in people with congenital limb deficiency or amputation in early childhood. *Brain*.

[29] Dunn D. S., Frank R. G., Elliott T. R. (2000). Social psychological issues in disability. *Handbook of Rehabilitation Psychology*.

[30] Gething L. (1991). Generality vs. specificity of attitudes towards people with disabilities. *The British Journal of Medical Psychology*.

[31] Burger H., Marinček Č. (1997). The life style of young persons after lower limb amputation caused by injury. *Prosthetics and Orthotics International*.

[32] Pezzin L. E., Dillingham T. R., MacKenzie E. J. (2000). Rehabilitation and the long-term outcomes of persons with trauma-related amputations. *Archives of Physical Medicine and Rehabilitation*.

[33] Schoppen T., Boonstra A., Groothoff J. W., de Vries J., Göeken L. N. H., Eisma W. H. (2001). Employment status, job characteristics, and work-related health experience of people with a lower limb amputation in the Netherlands. *Archives of Physical Medicine and Rehabilitation*.

[34] Krebs D. E., Fishman S. (1984). Characteristics of the child amputee population. *Journal of Pediatric Orthopaedics*.

[35] Norvell D. C., Turner A. P., Williams R. M., Hakimi K. N., Czerniecki J. M. (2011). Defining successful mobility after lower extremity amputation for complications of peripheral vascular disease and diabetes. *Journal of Vascular Surgery*.

[36] Frierson R. L., Lippmann S. B. (1987). Psychiatric consultation for acute amputees. Report of a ten-year experience. *Psychosomatics*.

[37] Thompson D. M., Haran D. (1985). Living with an amputation: the helper. *Social Science and Medicine*.

